# Sex differences in structural organization of motor systems and their dissociable links with repetitive/restricted behaviors in children with autism

**DOI:** 10.1186/s13229-015-0042-z

**Published:** 2015-09-04

**Authors:** Kaustubh Supekar, Vinod Menon

**Affiliations:** Department of Psychiatry & Behavioral Sciences, Stanford University School of Medicine, 401 Quarry Rd., Stanford, CA 94304-5719 USA; Department of Neurology and Neurological Sciences, Stanford University School of Medicine, Stanford, CA 94304 USA; Stanford Neurosciences Institute, Stanford University School of Medicine, Stanford, CA 94304 USA

**Keywords:** Sex differences, Children, Repetitive and restricted behavior, Motor, Brain structure

## Abstract

**Background:**

Autism spectrum disorder (ASD) is diagnosed much less often in females than males. Emerging behavioral accounts suggest that the clinical presentation of autism is different in females and males, yet research examining sex differences in core symptoms of autism in affected children has been limited. Additionally, to date, there have been no systematic attempts to characterize neuroanatomical differences underlying the distinct behavioral profiles observed in girls and boys with ASD. This is in part because extant ASD studies have included a small number of girls.

**Methods:**

Leveraging the National Database for Autism Research (NDAR), we first analyzed symptom severity in a large sample consisting of 128 ASD girls and 614 age- and IQ-matched ASD boys. We then examined symptom severity and structural imaging data using novel multivariate pattern analysis in a well-matched group of 25 ASD girls, 25 ASD boys, 19 typically developing (TD) girls, and 19 TD boys, obtained from the Autism Brain Imaging Data Exchange (ABIDE).

**Results:**

In both the NDAR and ABIDE datasets, girls, compared to boys, with ASD showed less severe repetitive/restricted behaviors (RRBs) and comparable deficits in the social and communication domains. In the ABIDE imaging dataset, gray matter (GM) patterns in the motor cortex, supplementary motor area (SMA), cerebellum, fusiform gyrus, and amygdala accurately discriminated girls and boys with ASD. This sex difference pattern was specific to ASD as the GM in these brain regions did not discriminate TD girls and boys. Moreover, GM in the motor cortex, SMA, and crus 1 subdivision of the cerebellum was correlated with RRB in girls whereas GM in the right putamen—the region that discriminated TD girls and boys—was correlated with RRB in boys.

**Conclusions:**

We found robust evidence for reduced levels of RRB in girls, compared to boys, with ASD, providing the strongest evidence to date for sex differences in a core phenotypic feature of childhood ASD. Sex differences in brain morphometry are prominent in the motor system and in areas that comprise the “social brain.” Notably, RRB severity is associated with sex differences in GM morphometry in distinct motor regions. Our findings provide novel insights into the neurobiology of sex differences in childhood autism.

**Electronic supplementary material:**

The online version of this article (doi:10.1186/s13229-015-0042-z) contains supplementary material, which is available to authorized users.

## Background

Autism spectrum disorder (ASD) is a highly heterogeneous neurodevelopmental disorder characterized by social impairments, communication difficulties, and repetitive/restricted behaviors (RRBs). One of the most consistent findings from epidemiological studies is that ASD is diagnosed less frequently in females than in males, with a ratio of 1 to 4 [1–4]. Despite the well-acknowledged sex differences in ASD prevalence rates and the anecdotal evidence suggesting that the clinical presentation of autism is different in females and males [5–8], research examining sex differences in core symptoms of autism in affected children has been limited. A better understanding of sex differences in core impairments in autism may inform the question of why there are fewer girls diagnosed with ASD than boys. For example, if girls with ASD, on average, exhibited less severe impairments than boys then that could cause delayed or missed diagnosis in girls. Apart from autism symptomatology, little is known about sex differences in brain organization in childhood ASD. This is in part because extant brain imaging studies have almost exclusively focused on boys or mixed gender samples involving a small number of girls, with a recent meta-analysis suggesting a large male bias of 8:1 in structural neuroimaging studies of autism [9]. Furthermore, how sex differences in neuroanatomy relate to sexual dimorphism in symptomatology is not known. This knowledge is critical not only for understanding the etiology of this heterogeneous disorder but also for understanding neuroprotective factors in girls [10].

The first aim of our study was to examine sex differences in the three core impairments that characterize childhood ASD. Findings from previous studies of sex differences in RRB have been largely inconsistent (Additional file 1: Table S1). Some studies have reported greater stereotypical play and RRB in males, compared to females, with ASD [11–13], while others have either found no sex differences [5, 14–16] or even greater abnormal motor disturbances in females [6]. Findings related to sex differences in social impairments have also been inconsistent (Additional file 1: Table S1). A few studies have reported greater social abilities and higher social-competence ratings in males, compared to females, with ASD [6, 15], other studies have observed no sex differences in non-verbal social behavior, social-cognitive behavior, and on the social domain of the Autism Diagnostic Interview, Revised (ADI-R) [14, 16, 17], and one study has reported greater impairments in group play and social problems in females, than males, with ASD [5]. Similarly, inconsistent findings have also been reported in the communication domain (Additional file 1: Table S1). Some studies have found that males with ASD had better language abilities than females [6], others have either found greater communication impairments and fewer current socio-communication difficulties in females than males with ASD [15, 18] or no sex differences in early social-communication skills and on the communication domain of the ADI-R or the ADOS [5, 19]. These discrepancies may be related to differences in symptom measures used, heterogeneity of the sample, and the wide age range studied. Importantly, the inconsistent nature of these findings could be attributed to small sample sizes that fail to capture the underlying heterogeneity of the disorder [8, 10]. Two recent studies attempted to address this problem by using meta-analytical [20] and data reuse (of the Simons Simplex Collection) [21] approaches. Although these studies were able to increase sample sizes beyond previous studies, findings may have been confounded by age and IQ differences as well as by differences in the clinical instruments used to assess ASD symptom severity and parent reports across datasets, as these factors were not controlled for [20, 21]. Accounting for these confounding factors is crucial due to the potential influence of age and IQ on autism symptom severity [22].

The second aim of our study was to investigate whether structural brain organization is different in girls and boys with ASD. In spite of increasing evidence that females with autism differ from males with the disorder across multiple levels, including genetics [23–25], proteomics [26, 27], and hormones [28], the number of studies examining sex differences in autism at the brain level is fairly small. The earliest among them examined 7 females and 38 males with autism and found no differences in cerebral enlargement between sexes [29]. A subsequent longitudinal study reported that females with autism showed a more pronounced abnormal brain overgrowth profile at the early stages of development (age range = 1.5–5 years) than males with autism, in a sample of 9 females and 32 males with autism [30]. A structural and diffusion tensor imaging study of white matter found sex differences in atypical corpus callosum neuroanatomy in preschool-aged children with ASD [31, 32]. In contrast, a recent diffusion tensor imaging study found no significant sex differences in neuroanatomy of major white-matter pathways in a sample of 12 male and 13 female adults with high-functioning autism [33]. Three recent studies focusing exclusively on females with autism reported greater regional gray matter (GM) volume in younger ASD females [31, 32] and lower GM densities in older ASD females [34]. A more recent study added ASD and neurotypical males to the female-only cohort and found minimal spatial overlap in atypical neuroanatomical features of autism in adult females and males [35]. Findings from these studies are, however, poorly replicated, likely because of the small number of participants, especially female participants, and the wide range in age and severity of ASD within these samples [9]. Importantly, many of these studies were conducted in adults with autism rather than children, which is problematic for a disorder with early life onset and variable developmental trajectory [10].

To address the first aim, we examined sex differences in social impairments, communication difficulties, and RRB in two well-characterized datasets consisting of (i) 128 girls with ASD and 614 age- and IQ-matched boys with ASD obtained from the open-access National Database for Autism Research (NDAR) [36] and (ii) 25 girls with ASD and 25 age- and IQ-matched boys with ASD obtained from the open-access multisite Autism Brain Imaging Data Exchange (ABIDE) [37]. On the basis of previous work [20, 21], we predicted that, compared to boys with ASD, girls with ASD would show reduced severity of RRB and comparable deficits in the social and communication domains in both datasets.

To address the second aim, we examined sex differences in neuroanatomy in the ABIDE dataset. Structural MRI data was not available for participants in the NDAR dataset. We combined voxel-based morphometry (VBM) [38] with univariate and multivariate pattern analysis (MVPA) [39] to determine GM regions that differ between girls and boys with ASD. Whereas univariate analyses reveal which particular brain regions differ on a relevant brain dimension (e.g., GM volume) between participant groups, multivariate analyses capture GM patterns that discriminate between two participant groups. MVPA techniques based on machine learning and cross-validation techniques provide greater sensitivity than the univariate approaches for detecting group differences [40]. Specifically, a multivariate analysis that takes into account spatial patterns in the data would be able to detect subtle changes in multiple brain areas that may accompany complex neuropsychiatric disorders such as autism, while the univariate would fail. This improved sensitivity is due to the consideration of spatial patterns of group differences, above and beyond those detectable at the individual voxel level. We hypothesized that, as with our previous study [40], MVPA would reveal multivoxel morphometric patterns that are different in girls and boys with ASD in multiple brain areas. To examine the specificity of sex differences in GM morphometry in ASD, we performed VBM with univariate and MVPA to identify GM regions that differ between typically developing (TD) girls and TD boys and then assessed whether the regions that could reliably distinguish girls with ASD from boys with ASD could also accurately distinguish TD girls from TD boys and vice versa. We predicted that MVPA would reveal GM morphometric patterns that are different in TD girls and TD boys. We further predicted that the patterns of GM morphometry ASD sex differences would be different from the normative sex difference patterns.

Finally, how sex differences in neuroanatomy might be related to sex differences in the behavioral phenotype of ASD is an open question in the field. To address this knowledge gap, we examined the relationship between the multivoxel brain morphometry patterns that are different in girls and boys with ASD and symptom severity in girls and boys with ASD. To investigate whether sex differences in the behavioral phenotype of ASD are linked to normative sex differences in neuroanatomy, we also explored the relationship between the multivoxel brain morphometry patterns that are different in TD girls and TD boys and symptom severity in girls and boys with ASD. We hypothesized that the brains of girls and boys with ASD would be structured in ways that contribute differently to behavioral impairments.

## Methods

### Participants

#### NDAR dataset

One hundred twenty-eight females with ASD (mean age: 9.83 years) and 614 males with ASD (mean age: 9.83 years) were included in this study. The subjects were identified from public domain research data repositories. Specifically, they were identified by querying NDAR (http://ndar.nih.gov). The query parameters were age 7 to 13 years, phenotype ASD, and IQ greater than 70. The query output was set to return age, gender, IQ, and phenotype along with scores on the Autism Diagnostic Interview, Revised (ADI-R). These query results yielded 3252 children with ASD. ADI-R scores or gender information was missing for 2510 children, and they were therefore not included in the study. Of the remaining 742 subjects, 128 were female and 614 were male. Structural MRI data, however, was not available for the subjects in this dataset.

Informed consent was obtained from each subject, and the study protocol was approved by the Institutional Review Board of the site where the data was collected.

#### ABIDE dataset

Twenty-five females with ASD (mean age: 10.3 years) and 25 males with ASD (mean age: 10.2 years) as well as 19 TD females (mean age: 10.2 years) and 19 TD males (mean age: 10.3 years) were included in this study. The subjects were identified from public domain research data repositories. Specifically, they were identified by querying ABIDE (http://fcon_1000.projects.nitrc.org/indi/abide). The query parameters were age 7 to 13 years, IQ greater than 70, and structural MRI present. The minimum age was set as 7 years because that was the age of the youngest participant made available by the ABIDE Consortium. Additionally, the maximum age was capped at 13 years to minimize the confounding effects of development and puberty status on our results, as done in extant studies of childhood autism [41]. The query output was set to return age, gender, IQ, and phenotype along with scores on the ADI-R. These query results yielded 25 females with ASD, 129 males with ASD, 31 TD females, and 116 TD males. These data were input to a customized subject-matching algorithm [42], which produced an age- and IQ-matched balanced gender and site sample consisting of 25 girls with ASD (mean age: 10.3 years) and 25 boys with ASD (mean age: 10.2 years) as well as 19 TD females (mean age: 10.2 years) and 19 TD males (mean age: 10.3 years). This aggregated well-matched dataset consisted of data from six sites/cohorts, including Kennedy Krieger Institute, New York University, Stanford University, University of California—Los Angeles, University of Michigan, and Yale University. Each site contributed equally to all four groups. For each of these sites, approval of the study protocol by the Institutional Review Board or an explicit waiver to provide fully anonymized data was required by the ABIDE Consortium before data contribution. A comprehensive list of all the review boards that approved the study is provided in the “Acknowledgements” section. Further, in accordance with the Health Insurance Portability and Accountability (HIPAA) guidelines, the ABIDE Consortium ensured that all the datasets were fully anonymized, with no protected health information included.

### Data analysis

#### Univariate autism symptoms analysis

To investigate sex differences in autism symptom severity, we compared (i) total scores on the ADI-R, (ii) scores on the social domain of the ADI-R, (iii) scores on the communication domain of the ADI-R, and (iv) scores on the RRB domain of the ADI-R, in ASD girls with those of ASD boys, using two-sample *t*-tests.

#### Multivariate autism symptoms-based classification analysis

In addition to univariate analysis, symptom severity data were subjected to a multivariate classification analysis. Briefly, multivariate classification analysis was performed to determine whether scores on various ADI-R domains taken together could discriminate girls with ASD from boys with ASD. Scores on the social, communication, and RRB domains of the ADI-R were used as the input (features) to a classifier. The classifier distinguishes girls with ASD from boys with ASD by making a classification decision based on the value of the linear combination of these features. A sparsity-promoting linear classifier (GLMNet: http://cran.r-project.org/web/packages/glmnet/) that was best suited for our goals of classification based on an identifying feature set that accurately discriminated the two groups was used in our analysis. Leave-one-out cross-validation (LOOCV) was used to measure the performance of the classifier in distinguishing girls with ASD from boys with ASD. In LOOCV, one single observation is used for testing the classifier that is trained using the remaining observations. This process is repeated such that every observation is used once for testing purposes.

#### Voxel-based morphometry

Brain morphometry was assessed using the optimized voxel-based morphometry (VBM) method [38] performed with the VBM5 toolbox (http://dbm.neuro.uni-jena.de/vbm). Prior to analyses, the structural images were resliced with trilinear interpolation to isotropic 1 × 1 × 1 voxels and aligned to conventional anterior commissure (AC)-posterior commissure (PC) space using manually identified landmarks, including the AC, the PC, and the mid-sagittal plane. The resliced images were spatially normalized to the Montreal Neurological Institute (MNI) stereotactic space. Spatial transformation was nonlinear with warping regularization = 1; warp frequency cutoff = 25. The spatially normalized images were then segmented into GM, white matter (WM), and cerebrospinal fluid (CSF) compartments, with a modified mixture model cluster analysis technique [43] with the following parameters: bias regularization = 0.0001, bias full width at half maximum cutoff = 70 mm, and sampling distance = 3. No tissue priors were used for segmentation. Voxel values were modulated by the Jacobian determinants derived from the spatial normalization such that the areas that were expanded during warping were proportionally reduced in intensity. The investigators used modulation for nonlinear effects only (while the warping included both an affine and a nonlinear component). When using modulated images for performing subsequent group comparisons, the inference is made on measures of volume rather than tissue concentration (density). The use of modulation for nonlinear but not affine effects ensures that the statistical comparisons are made on relative (e.g., while controlling for overall brain size) rather than absolute volumes. The segmented (modulated) images for white and gray matter were smoothed with an isotropic Gaussian kernel (10 mm full width at half maximum).

### Univariate morphometric analysis

Univariate two-sample *t*-tests were applied to smoothed modulated GM images to find brain regions that discriminated (i) girls with ASD from boys with ASD and (ii) TD girls from TD boys. Additionally, a group (ASD, TD) by sex (male, female) ANOVA was applied to smoothed modulated GM images to determine how ASD diagnostic status moderates normative sex differences in the brain. In each of the aforementioned univariate analyses, age and site were included as covariates of no interest.

### Multivariate morphometric pattern-based classification analysis

In addition to univariate analysis, an MVPA method [40, 44] was applied to smoothed modulated GM images to find brain regions that discriminated (i) girls with ASD from boys with ASD and (ii) TD girls from TD boys. The MVPA procedure is illustrated in Additional file 2: Figure S1. MVPA analysis was performed using LIBSVM software (http://www.csie.ntu.edu.tw/~cjlin/libsvm/). Inputs into the MVPA were the smoothed GM maps computed from the VBM analyses. Age and site were included as covariates of no interest. The MVPA method uses a nonlinear classifier based on support-vector machine (SVM) algorithms with radial basis function (RBF) kernels. Briefly, at each voxel *v*_*i*_, a 3 × 3 × 3 neighborhood (“searchlight”) centered at *v*_*i*_ was defined. The spatial pattern of voxels in this block was defined by a 27-dimensional vector. For the nonlinear SVM classifier, two parameters were specified, *C* (regularization) and *α* (parameter for RBF kernel), at each searchlight position. Optimal values of *C* and *α* and the generalizability of the classifier were estimated at each searchlight position by using a combination of grid search and cross-validation procedures. In earlier approaches, linear SVM was used, and the free parameter, *C*, was arbitrarily set. In the current work, however, free parameters (*C* and *α*) were optimized based on the data, thereby designing an optimal classifier. In the *M*-fold (here *M* = 10) cross-validation procedure, the data were randomly divided into *M*-folds. *M* - 1 folds were used for training the classifier, and the remaining fold was used for testing. This procedure was repeated *M* times wherein a different fold was left out for testing each time. Class labels of the test data were estimated at each fold, and average classification accuracy was computed for each fold, termed cross-validation accuracy (CA). The optimal parameters were found by grid searching the parameter space and selecting the pair of values (*C*, *α*) at which the *M*-fold cross-validation accuracy was maximum. To search for a wide range of values, we varied the values of *C* and *α* from 0.125 to 32 in steps of 2 (0.125, 0.25, 0.5, 2, 16, 32). The resulting 3-D map of cross-validation accuracy at every voxel was used to detect brain regions that discriminated between the two participant groups. Under the null hypothesis that there is no difference between the two groups, the CAs were assumed to follow the binomial distribution *B*_*i*_(*N*, *p*). The statistical maps were thresholded as follows: height 0.001, Family-wise Error (FWE) corrected, and extent 40 voxels (0.01). These extent thresholds were determined using Monte-Carlo simulations on the GM mask. Monte-Carlo simulations were implemented in Matlab using methods similar to the AlphaSim procedure in the Analysis of Functional Neuroimages (AFNI) software.

### Multivariate support vector regression analysis: relationship between morphometry and autism symptom severity

After using MVPA to identify the GM regions producing the highest classification accuracies for discriminating girls with ASD from boys with ASD, we looked for relationships between the morphometry in the identified brain regions and symptom severity based on diagnostic criteria (ADI-R scores) in each group. This was accomplished by conducting a support-vector regression (SVR) analysis using regional GM morphometry as the independent variable and symptom severity, as measured using ADI-R diagnostic algorithm, as the dependent variable. In contrast to the conventional univariate correlation analysis, SVR allows examination of relationships between multiple independent variables with a dependent variable. Briefly, we used SVR analysis to examine the relationships between GM volume pattern across multiple contiguous voxels belonging to a brain region of interest and ASD symptom severity. The multivariate nature of our SVR analysis that takes into account spatial patterns in the data would detect a subtle pattern across multiple brain areas—that may accompany complex neuropsychiatric disorders such as autism—that predicts behavior, while the univariate would fail.

In the SVR analysis, we focused on brain regions that discriminated girls with ASD from boys with ASD. Briefly, ROIs were selected from the ASD girls’ versus ASD boys’ classification map. After visually selecting a voxel with the highest classification accuracy within each cluster on the classification map, the ROIs were constructed by drawing spheres with centers as the seed point and a radius of 8 mm. Age and site were included as covariates of no interest. We used SVR with the default settings of *C* = 1 and nu = 0.05, as implemented in the LIBSVM Toolbox (http://www.csie.ntu.edu.tw/~cjlin/libsvm/). For each ROI, we first estimated *R*^2^ using the leave-one-out cross-validation procedure. Each sample was designated the test sample in turns while the remaining samples were used to train the SVR predictor. The decision function derived from the training sample was then used to make a real-valued prediction about the test sample. *R*^2^ was computed based on the observed and predicted values. Finally, the statistical significance of the SVR model was assessed using non-parametric analysis. The empirical null distribution of *R*^2^ was estimated by generating 1000 surrogate datasets under the null hypothesis that there was no association between regional GM morphometry and symptom severity. Each surrogate dataset *D*_*i*_ of size equal to the observed dataset was generated by permuting the labels (symptom severity scores) on the observed data points. The SVR model was fitted to predict labels of each surrogate dataset *D*_*i*_. *R*_*i*_^2^ was computed using the actual labels of *D*_*i*_ and predicted labels. This procedure produces a null distribution of *R*^2^ of the SVR model. The statistical significance (*p* value) of the model was then determined by counting the number of *R*_*i*_^2^ greater than *R*^2^ and dividing that count by the number of *D*_*i*_ (=1000). We corrected for multiple comparisons using a false discovery rate (FDR) control procedure.

## Results

### Demographic and neuropsychological profile

In the NDAR dataset, girls and boys with ASD did not differ in age (*p* = 0.79, *t*_(740)_ = −0.27) or IQ (*p* = 0.47, *t*_(740)_ = 0.70).

In the ABIDE dataset, a group (ASD, TD) by sex (male, female) ANOVA revealed no significant effect of group, nor of gender, nor their interaction, on age, IQ, and handedness (all *p*’s > 0.19) (Table 1).

**Table 1:** Demographic and neuropsychological measures in ASD boys, ASD girls, TD boys, and TD girls of the ABIDE cohort

Measure	ASD boys	ASD girls	TD boys	TD girls
*N*	25	25	19	19
Age (years)	10.19 (±0.30)	10.31 (±0.30)	10.34 (±0.35)	10.24 (±0.35)
	(Range: 7.20–12.37)	(Range: 8.09–12.79)	(Range: 8.39–12.81)	(Range: 8.50–12.73)
Handedness^a^	18R/2L	19R/1L	15R/0L	14R/1L
Full IQ	102.16 (±3.02)	103.62 (±2.82)	105.05 (±2.96)	111.63 (±3.82)
	(Range: 78–131)	(Range: 88–134)	(Range: 85–142)	(Range: 80–129)

The groups did not differ in age, handedness, or IQ

^a^Handedness information was not available for 10 ASD (5 M/5 F) and 8 TD (4 M/4 F) participants

### Autism symptoms

**Fig. 1:**
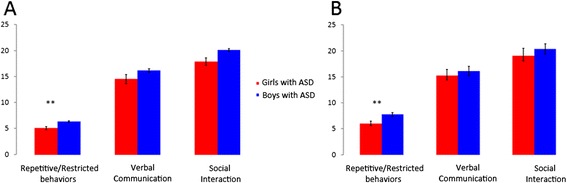
Sex differences in core impairments in childhood autism. **a** In the NDAR dataset, girls with ASD showed less severe repetitive and restricted behavior, as measured by scores on the repetitive/restricted behavior domain of the ADI-R. There were no sex differences in scores on the social domain of the ADI-R as well as the communication domain of the ADI-R. **b** In the ABIDE dataset, similar to the results observed in the NDAR dataset, girls with ASD showed less severe repetitive and restricted behavior, as measured by scores on the repetitive/restricted behavior domain of the ADI-R. There were no sex differences in scores on the social domain of the ADI-R as well as the communication domain of the ADI-R

In the NDAR dataset, girls and boys did not differ in overall severity of ASD, as measured by total scores on the ADI-R (*p* = 0.12, *t*_(740)_ = −1.15). Also, there were no sex differences in scores on the social domain of the ADI-R (*p* = 0.28, *t*_(740)_ = −1.09) nor on the communication domain of the ADI-R (*p* = 0.12, *t*_(740)_ = −1.15). However, girls with ASD showed less severe RRB, as measured by the ADI-R (*p* < < 0.01, *t*_(740)_ = −5.19) (Fig. 1a). To further demonstrate the robustness of our findings, we investigated whether scores on various ADI-R domains taken together could discriminate girls with ASD from boys with ASD, using a multivariate sparsity-promoting linear classifier. This analysis revealed that girls with ASD could be distinguished from boys with ASD on the basis of their ADI-R domain scores with an accuracy of 94 %. Notably, the most significant feature that discriminated the two groups was the ADI-R RRB domain score. The ADI-R social as well as communication domain scores were not significant (zero), i.e., they did not contribute to the discrimination of girls and boys with ASD. These results further highlight the specificity of our finding of sex differences in RRB in childhood autism.

In the ABIDE dataset, similar to the results observed in the NDAR dataset, girls and boys did not differ in overall severity of ASD (*p* = 0.24, *t*_(45)_ = −1.19), as measured by total scores on the ADI-R. Also, there were no sex differences in scores on the social domain of the ADI-R (*p* = 0.47, *t*_(45)_ = −0.73) nor on the communication domain of the ADI-R (*p* = 0.57, *t*_(45)_ = −0.57). However, girls with ASD showed less severe repetitive/restricted behaviors, as measured by scores on the RRB domain of the ADI-R (*p* < 0.01, *t*_(45)_ = −2.78) (Fig. 1b). Multivariate classification analysis revealed results similar to those observed in the NDAR dataset. Namely, girls with ASD could be distinguished from boys with ASD on the basis of their ADI-R domain scores with an accuracy of 89 %. Notably, the most significant feature that discriminated the two groups was the ADI-R RRB domain score. The ADI-R social as well as communication domain scores were not significant (zero), i.e., they did not contribute to the discrimination of girls and boys with ASD.

### Univariate morphometric analysis: girls with ASD vs. boys with ASD

To delineate neural markers that underlie the unique symptom profile in girls with ASD, we compared brain structure in girls with ASD and boys with ASD. Using univariate analysis, we found no differences in GM volume between girls with ASD and boys with ASD.

### Multivariate morphometric pattern-based classification analysis: girls with ASD vs. boys with ASD

**Fig. 2:**
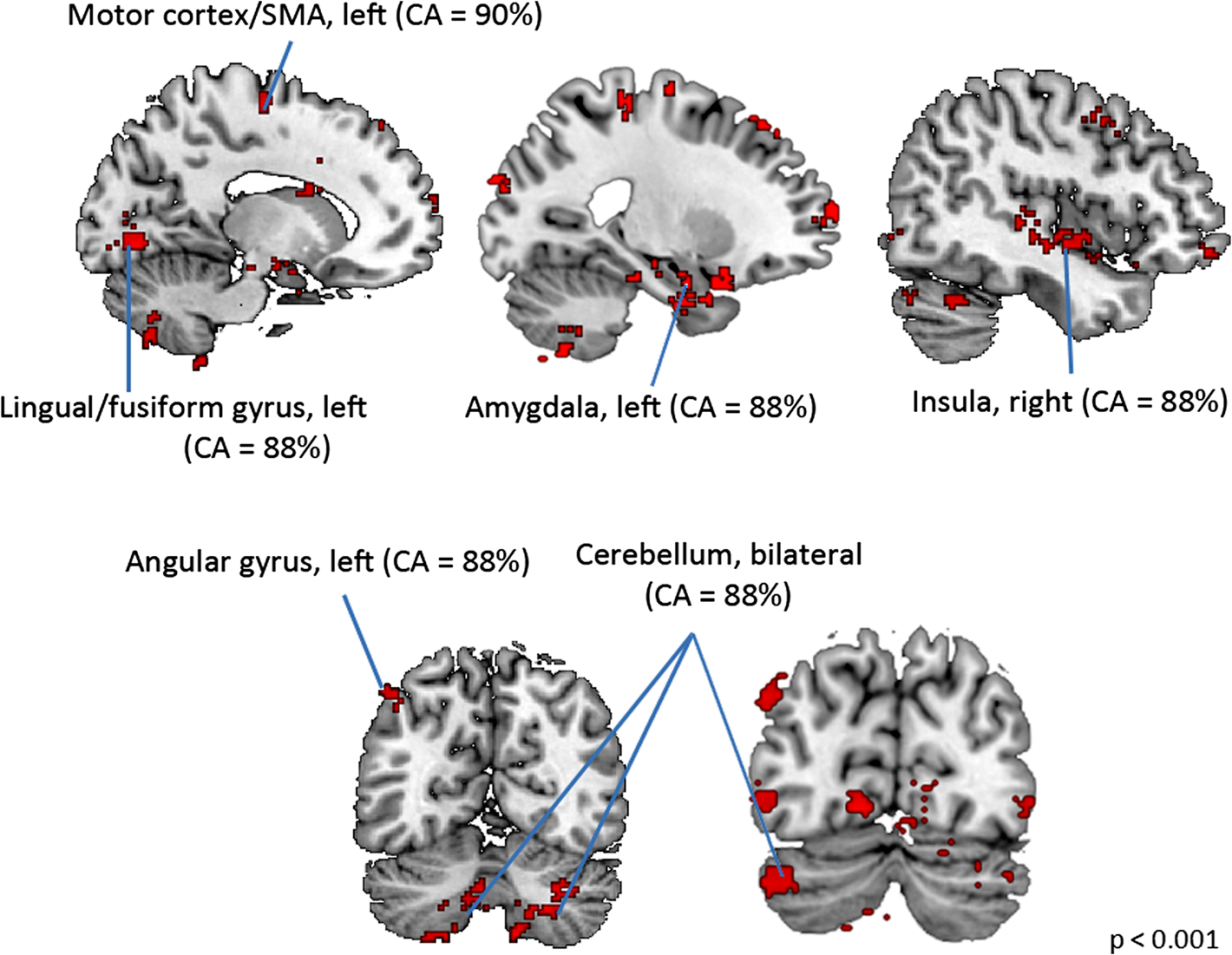
Sex differences in brain morphometry in childhood autism. Girls and boys with ASD showed significant differences in brain structure. Notably, brain areas which showed sex differences in ASD fell into two general functional systems: the motor system and systems that form part of the “social brain.” These brain areas include the left motor cortex, left SMA, left lingual/fusiform gyrus, left angular gyrus, right insula, bilateral cerebellum, and bilateral amygdala. They showed high classification accuracies (CA > 85 %) for distinguishing girls from boys with ASD. CA value given for a set of contiguous voxels corresponds to the highest classification accuracy among those voxels

Using MVPA analysis (Additional file 2: Figure S1), we found that the GM in several cortical and subcortical regions could differentiate girls and boys with ASD. Notably, GM volume in the left motor cortex, left supplementary motor area (SMA), left lingual/fusiform gyrus, left angular gyrus, right insula, bilateral cerebellum, and bilateral amygdala (height *p* < 0.001, FWE corrected, extent *p* < 0.01; Table 2) showed high accuracies (85–90 %) for distinguishing girls from boys with ASD (Fig. 2).

**Table 2:** Gray matter morphometry girls with ASD vs. boys with ASD classification peaks

	MNI coordinates				
Region	*X*	*Y*	*Z*	Cluster size	Classification accuracy (%)
L motor cortex/SMA	−14	−14	64	95	90
R amygdala	28	2	−34	190	88
L lingual/fusiform gyrus	−12	−76	−6	103	88
L angular gyrus	−46	−62	52	179	88
R insula	44	−12	−2	499	87
R cerebellum	12	−64	−54	295	85
L cerebellum	−18	−72	−56	212	85
L amygdala	−20	−2	−26	312	85

*SMA* supplementary motor area

### Univariate morphometric analysis: TD girls vs. TD boys

Sex differences in brain structure are prominent in typically developing individuals [45]. To address the specificity of our findings on sex differences in children with ASD, we first compared brain structure between TD girls and TD boys. Using univariate analysis, we found no differences in the GM volume between TD girls and TD boys. Next, we performed a univariate group (ASD, TD) *by* gender (females, males) ANOVA analysis of GM volume, which revealed no significant effect of group, gender, or their interaction.

### Multivariate morphometric pattern-based classification analysis: TD girls vs. TD boys

Using MVPA analysis, consistent with evidence from previous structural neuroimaging studies of normative sex differences in the brain structure of children [45], we found that GM in several cortical and subcortical regions could discriminate TD girls from TD boys. Notably, GM volume in the right postcentral gyrus, left parahippocampus, right lateral occipital cortex, right putamen, and bilateral cerebellum (*p* < 0.001) showed high accuracies (85–90 %) for distinguishing TD girls from TD boys.

To investigate how ASD diagnostic status moderates these normative sex differences in multivariate GM morphometry, we assessed whether brain areas that showed sex differences in brain morphometry in TD children were also different in their ASD peers. Specifically, we asked whether regions that could reliably distinguish TD girls from TD boys could also accurately distinguish girls with ASD from boys with ASD. We found that, except for the cerebellum, none of the regions examined could accurately differentiate ASD girls from ASD boys.

Additionally, we assessed whether brain areas that showed sex differences in brain morphometry in ASD were also different in their TD peers. Specifically, we asked whether regions that could reliably distinguish girls with ASD from boys with ASD could also accurately distinguish TD girls from TD boys. We found that, except for the cerebellum, none of the regions examined could accurately differentiate TD girls from TD boys. These results point to the unique spatial pattern of sex differences in children with ASD.

### Multivariate support vector regression analysis: relationship between morphometry and autism symptom severity

**Fig. 3:**
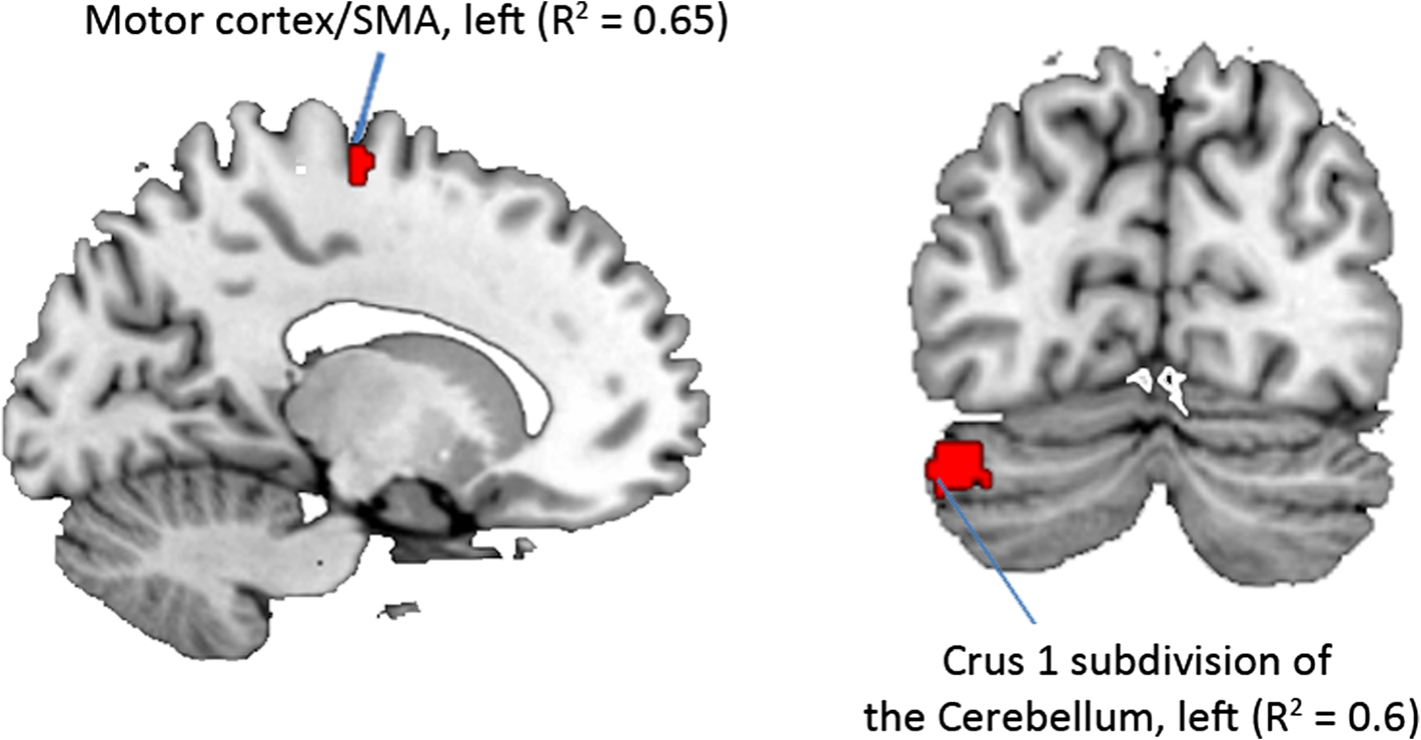
Relationship between sex differences in core impairments and brain morphometry in childhood autism. Gray matter volume in the motor cortex, SMA, and crus 1 subdivision of the cerebellum was correlated with scores on the repetitive/restrictive domain of the ADI-R

SVR analysis using multivariate GM morphometry of regions that discriminated boys with ASD from girls with ASD as the independent variable and symptom severity, as measured by the ADI-R diagnostic algorithm, as the dependent variable, revealed that the GM volume in the motor cortex, SMA, and crus 1 subdivision of the cerebellum were correlated with scores on the RRB domain of the ADI-R in girls with ASD (*p <* 0.05; Fig. 3*).* No such relationship was observed in boys or for the social and communication domains in either girls or boys (all *p*’s > 0.48).

To further examine the neural correlates of motor deficits in boys with ASD, we performed a SVR analysis in boys with ASD using GM morphometry of regions that discriminated TD boys from TD girls, as the independent variable, and symptom severity, as measured by the ADI-R diagnostic algorithm, as the dependent variable. We found that the GM volume in the right putamen was correlated with scores on the RRB domain of the ADI-R (*p <* 0.05). No such relationship was observed in girls or for the social and communication domains in either boys or girls (all *p*’s > 0.64).

## Discussion

Leveraging NDAR and ABIDE, two open-access large-scale databases, we found robust evidence for reduced levels of repetitive/restricted behaviors (RRBs) in girls, compared to boys, with ASD, providing the strongest evidence to date for sex differences in a core phenotypic feature of childhood ASD. Furthermore, analysis of neuroanatomical data from the ABIDE dataset revealed, for the first time, that girls and boys with ASD differ in the organization of cortical and subcortical motor systems and that RRB severity is associated with sex differences in GM morphometry in distinct motor systems. Collectively, these findings, as elaborated below, provide new insights into the neurobiology of sex differences in childhood autism.

### Sex differences in repetitive/restricted behaviors in childhood autism

We found a specific profile of sex differences in the autism behavioral phenotype. Girls with ASD showed less RRB as compared to boys with ASD, but the two groups did not differ in the social behavior and communication domains. There were no sex differences in the overall symptom severity suggesting that girls and boys diagnosed with the disorder were similarly autistic. This pattern was observed in the larger NDAR dataset with 742 girls and boys and replicated in the smaller ABIDE dataset with 50 girls and boys with ASD. Our findings help resolve contradictory findings in the literature on sex differences in the core triad of autism symptoms. Crucially, by using two well-characterized datasets of high-functioning girls and boys who were well-matched on age and IQ and by using a single instrument to measure autism severity across datasets, we were able to overcome multiple limitations of previous studies [20, 21].

Our findings suggest a potential factor that may contribute to the relatively low proportion of females with ASD. Among the three core autism phenotypes, repetitive/restricted behaviors are the most overt and noticeable feature that flags a potential case of the disorder [10, 21]. Our findings raise the possibility that girls with less prominent RRB may miss being tested for ASD or get misclassified as having social communication disorder [46]. On the other hand, boys with more pronounced RRB may show more false positives for ASD, given that repetitive/restricted behaviors are not specific to children with ASD and are also observed in other neurodevelopmental disorders [20, 47]. Regardless of the potential impact on diagnosis, our findings point to a need for further research on the development of clinical instruments that are better tailored towards autism in females [48]. Additionally, with the emerging view that RRB through its purported association with language deficits may serve as an endophenotype of ASD, future work should examine the link between the sex differences in RRB and the lack of sex differences in communication impairments reported here, and sex-specific risk genes in ASD [49].

### Sex differences in brain morphometry in childhood autism

Girls and boys with ASD also showed significant differences in brain structure. MVPA revealed that GM morphometric patterns in girls with ASD are differently organized than in boys with ASD. In contrast, univariate analysis showed no GM differences between girls and boys with ASD, further highlighting the power of multivariate approaches in uncovering subtle changes in multiple brain areas that may accompany complex neuropsychiatric disorders such as autism [40]. Specifically, MVPA revealed that GM in multiple distributed cortical and subcortical regions significantly differentiated girls and boys with ASD with high classification accuracies. Briefly, brain regions with high classification accuracies can be interpreted as those in which there is information that can be gleaned from GM morphometric patterns across neighboring voxels that can be used to assign a particular individual to a group—in our case, girls with ASD or boys with ASD [39]. Extending this interpretation to a group difference point of view, brain regions with high classification accuracies are those in which the GM multivoxel morphometry pattern is significantly different between girls with ASD and boys with ASD [40]. It is noteworthy that brain areas which showed sex differences in ASD could be classified into two general functional systems: the motor system and systems that form part of the “social brain” [50, 51].

### Specificity of sex differences in brain morphometry in childhood autism

Sex differences in the brain structure are prominent in typically developing individuals [45]. Consistent with evidence from several previous structural neuroimaging studies of typical children [45], we found normative sex differences in the right postcentral gyrus, left parahippocampus, right lateral occipital cortex, right putamen, and bilateral cerebellum. We found that, except for the cerebellum, none of these regions could accurately differentiate ASD girls from ASD boys. To further examine the influence of ASD diagnostic status on normative sex differences in GM morphometry, we assessed whether brain areas that showed sex differences in brain morphometry in ASD were also different in their TD peers. We found that, except for the cerebellum, none of the regions examined could accurately differentiate TD girls from TD boys. These results suggest that ASD diagnostic status moderates normative sex differences in multivariate GM morphometry and further highlight the unique profile of neuroanatomical sex differences—in the motor system and the “social brain”—in children with ASD.

### Sex differences in morphometry of motor system and links to repetitive/restricted behaviors

MVPA revealed that GM in the motor system significantly differentiated girls and boys with ASD with high classification accuracies. Specifically, the highest classification accuracies (85–90 %) were observed in the motor cortex, SMA, and the crus 1 subdivision of the cerebellum, regions involved in motor planning and execution [52]. A recent meta-analysis of structural neuroimaging studies of ASD found evidence for significant GM abnormalities in these motor areas [53]. A more recent study observed that childhood ASD is associated with atypical morphology of cortical areas that are critical to motor control and learning [54]. Motor impairments are prominent in infants, children, and adults with the disorder [55] and have been associated with repetitive behaviors, a core feature of the ASD phenotype [56, 57]. Reduced cerebellar GM volume has been reported to be associated with increased stereotyped and repetitive movements [56, 57]. Functional connectivity studies have revealed that children with ASD compared to their TD peers exhibit reduced functional connectivity in the motor control network during finger sequencing [58]. Connectivity within and between functional subregions of the precentral gyrus, particularly involving the dorsomedial subregion, has also been observed to be related to ASD diagnosis and traits [59]. Our findings extend these results by providing novel evidence that the morphometry of the motor system is different in girls and boys with ASD.

We then examined the hypothesis that the sex differences detected in the motor system would be related to the observed differences in the RRB between girls with ASD and boys with ASD. We first focused on brain areas that showed sex differences in ASD.

Based on brain regions that showed sex differences in ASD, we found that the GM morphometry in the motor cortex, SMA, and cerebellum was correlated with scores on the RRB domain of the ADI-R. They were not correlated with scores on the social behavior and communication scores of the ADI-R, indicating domain-specific effects. These relations were observed in girls, but not in boys with ASD.

To clarify these findings in the context of normative/fundamental sex differences in TD individuals, we conducted additional analyses focusing on sex differences in the TD group. Based on regions that showed sex differences in TD children, we found that GM morphometry in the right putamen—a brain region consistently showed in multiple studies including ours to have normative sex differences in its GM morphometry—was correlated with scores on the RRB domain of the ADI-R. These relations were observed in boys, but not in girls with ASD. No such relations were found with respect to the social behavior and communication domain of the ADI-R in either boys or girls.

These results indicate that different components of the motor system may contribute to individual differences and heterogeneity of motor deficits in girls and boys with the disorder. In sum, our findings support the idea that that the observed sex differences in the ASD phenotype are linked to dimorphic brain structure in ASD. The neurobiological mechanisms underlying this dimorphism and its behavioral implications remain to be investigated.

### Sex differences in morphometry of “social brain” areas

In addition to ASD sex differences in brain areas involved in motor function, we found high classification accuracies of GM in several regions including the fusiform gyrus, angular gyrus, amygdala, and insula. These brain regions are commonly activated during various tasks involving face processing, recognizing emotions from faces, theory of mind, and visceral responses to social stimuli and are part of a system collectively referred to as the “social brain” [51]. Previous research in mixed groups of females and males with ASD has identified aberrations in each of these brain areas. A meta-analysis of 24 voxel-based morphometry studies found robust evidence for GM decreases in the amygdala complex in individuals with ASD compared to healthy controls [9]. Structural abnormalities in the anterior insula as well as the fusiform gyrus have also been similarly reported in individuals with ASD [60–63]. A recent fMRI investigation revealed that functional connectivity abnormalities underlying ASD were most pronounced between regions of the social brain [50]. Taken together, these results point to sex differences in several key areas that form part of the “social brain”.

However, morphometric patterns in the fusiform gyrus, amygdala, and insula regions that showed sex differences were not related to the severity of social symptoms in either group. Further studies are needed to investigate the functional and behavioral implications of morphometric differences in social brain areas in girls and boys with ASD. One potential avenue for investigation is anecdotal reports suggesting that females with ASD may be able to mask social difficulties by imitation and other compensatory strategies [10].

### Limitations

The study has four limitations that merit discussion. First, as in extant empirical studies, our sample was limited to children with high-functioning ASD. Further research is needed to investigate whether sex differences are also present in more severely affected individuals. Second, the female as well as male ASD participants included in our study received their diagnosis using the same instrument—in our case, the ADI-R. Given that the instrument itself is thought to be male biased, further studies utilizing sex-specific behavioral measures of ASD are needed to investigate whether the study findings are confounded by current diagnostic procedures. Third, in our study, RRB was measured using the historical ADI-R diagnostic algorithm-based scores, the only symptom severity values made available for all participants in the NDAR and ABIDE cohorts. Further research is needed to investigate how the observed sex differences in neuroanatomy relate to current ADI-R RRB scores, current ADI-R RRB subscales scores-repetitive, sensory motor behaviors (RSM), insistence on sameness (IS) and circumscribed interests, and/or other measures of RRB including the Repetitive Behaviors Scale-Revised (RBS-R). Fourth, the VBM approach used in the study only characterizes volume. Future work is required to examine sex differences in the cortical surface area and cortical thickness—the two components of volume.

## Conclusions

Our findings not only provide evidence for distinct behavioral phenotypes in girls with ASD, compared to boys, but also link behavioral differences to brain structure. Importantly, the severity of repetitive/restricted behaviors is lower in girls with ASD and is associated with sex differences in GM morphometry in cortical and cerebellar regions involved in motor control. Our findings indicate that the brains of girls with ASD are structured differently from those of boys and that some of these differences are linked to sex differences in behavioral impairments.

## Availability of supporting data

The data sets supporting the results of this article are available in the NDAR and ABIDE repositories.

## Additional files

Additional file 1: Table S1. Summary of findings from previous studies that examined sex differences in the three core impairments—RRB, social, and communication—that characterize ASD. Additional file 2: Figure S1. Multivariate pattern analysis (MVPA) to investigate sex differences in structural brain morphometry. MVPA analysis was performed using LIBSVM software (http://www.csie.ntu.edu.tw/~cjlin/libsvm/). Inputs into the MVPA were the smoothed GM maps computed from the VBM analyses. The MVPA method uses a nonlinear classifier based on support-vector machine algorithms with radial basis function (RBF) kernels. Briefly, at each voxel *v*_*i*_, a 3 × 3 × 3 neighborhood (“searchlight”) centered at *v*_*i*_ was defined. The spatial pattern of voxels in this block was defined by a 27-dimensional vector, which was inputted into the classifier. Classifier performance for *v*_*i*_ was computed using an *M*-fold cross-validation procedure. In the *M*-fold (here *M* = 10) cross-validation procedure, the data were randomly divided into *M*-folds. *M*-1 folds were used for training the classifier, and the remaining fold was used for testing. This procedure was repeated *M* times wherein a different fold was left out for testing each time. Class labels of the test data were estimated at each fold, and average classification accuracy was computed for each fold, termed cross-validation accuracy (CA).

## Abbreviations

ABIDE: Autism Brain Imaging Data Exchange
AC: anterior commissure
ADI-R: Autism Diagnostic Interview, Revised
ASD: autism spectrum disorder
CA: cross-validation accuracy
CSF: cerebrospinal fluid
GM: gray matter
MNI: Montreal Neurological Institute
MVPA: multivariate pattern analysis
NDAR: National Database for Autism Research
PC: posterior commissure
RBF: radial basis function
RRBs: repetitive/restricted behaviors
SMA: supplementary motor area
SVM: support-vector machine
SVR: support-vector regression
TD: typically developing
VBM: voxel-based morphometry
WM: white matter
