# Supplementary material for: Sex differences in structural organization of motor systems and their dissociable links with repetitive/restricted behaviors in children with autism
Source: Mol Autism. 2015 Sep 4;6:50. doi: 10.1186/s13229-015-0042-z (PMC4559968; doi:10.1186/s13229-015-0042-z)
Supplement: Additional file 1: Table S1. — Summary of findings from previous studies that examined sex differences in the three core impairments—RRB, social, and communication—that characterize ASD. [file 13229_2015_42_MOESM1_ESM.doc]

**Additional file 1**

**Table S1.** Summary of findings from previous studies that examined sex differences in the three core impairments – RRB, social, and communication – that characterize ASD.

| **Impairment**  **Domain** | **Study** | **N**  **ASD F ASD M** | | **Age (years)**  **ASD F ASD M** | | **Instrument** | **Finding** |
| --- | --- | --- | --- | --- | --- | --- | --- |
|  |  |  |  |  |  |  |  |
| **RRB** | Tsai et.al.  Lord et.al.  Bolte et.al.  Banach et.al.  Carter et.al.  McLennan et.al.  Holtman et.al.  Hattier et.al. | 24  91  35  48  22  21  23  63 | 78  384  21  108  68  21  23  77 | 6  3 – 8  14  9  2  6 – 36  2 – 21  49 | 6  3 - 8  14  9  2  6 - 36  2 - 21  49 | -  PEP, CARS  ADI-R, ADOS  ADI-R  Mullen Scale  ADI  ADI-R, ADOS  DASH-II | ASD F **>** ASD M  ASD F < ASD M  ASD F < ASD M  ASD F < > ASD M  ASD F < > ASD M  ASD F < > ASD M  ASD F < > ASD M  ASD F < ASD M |
|  |  |  |  |  |  |  |  |
| **Social Impairment** | Tsai et.al.  Lord et.al.  Banach et.al.  Carter et.al.  McLennan et.al.  Holtman et.al.  Lai et.al.  Lai et.al. | 24  91  48  22  21  23  32  54 | 78  384  108  68  21  23  32  54 | 6  3 – 8  9  2  6 – 36  2 – 21  28  5 | 6  3 - 8  9  2  6 - 36  2 - 21  27  5 | -  PEP, CARS  ADI-R  Mullen Scale  ADI  ADI-R, ADOS  ADI-R, ADOS  Eyes Test | ASD F **>** ASD M  ASD F < > ASD M  ASD F < > ASD M  ASD F > ASD M  ASD F < > ASD M  ASD F > ASD M  ASD F < ASD M  ASD F < > ASD M |
|  |  |  |  |  |  |  |  |
| **Communication**  **Difficulties** | Tsai et.al.  Carter et.al.  McLennan et.al.  Holtman et.al.  Lai et.al. | 24  22  21  23  32 | 78  68  21  23  32 | 6  2  6 – 36  2 – 21  28 | 6  2  6 - 36  2 - 21  27 | -  Mullen Scale  ADI  ADI-R, ADOS  ADI-R, ADOS | ASD F **>** ASD M  ASD F > ASD M  ASD F < ASD M  ASD F< > ASD M  ASD F < ASD M |
|  |  |  |  |  |  |  |  |

PEP: Psychoeducational Profile; CARS: Childhood Autism Ratings Scale; DASH-II - Diagnostic Assessment for the Severely Handicapped-II;
