# Supplementary figures and images for: Sex differences in structural organization of motor systems and their dissociable links with repetitive/restricted behaviors in children with autism
Source: Mol Autism. 2015 Sep 4;6:50. doi: 10.1186/s13229-015-0042-z (PMC4559968; doi:10.1186/s13229-015-0042-z)

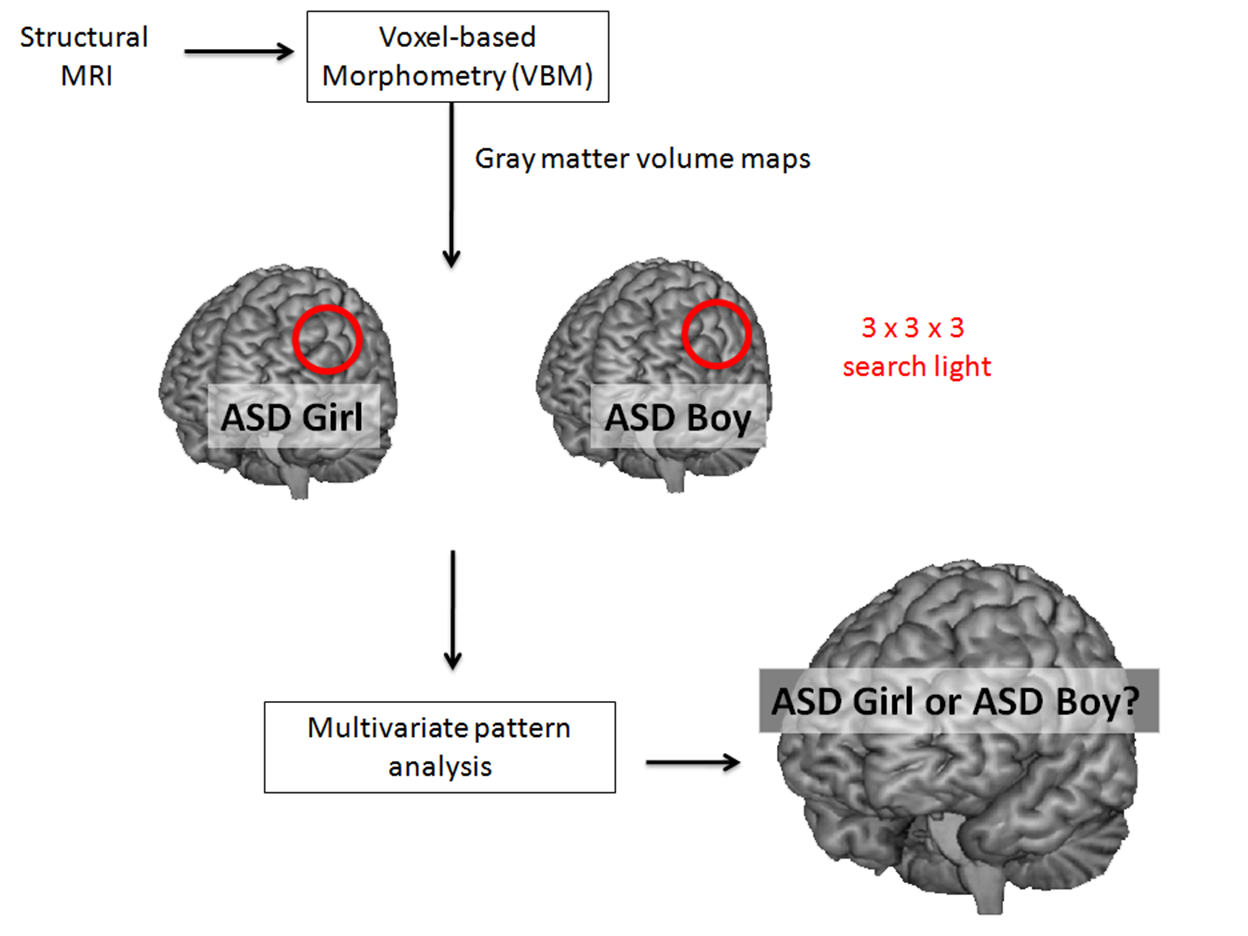

Supplement: Additional file 2: Figure S1. — Multivariate pattern analysis (MVPA) to investigate sex differences in structural brain morphometry. MVPA analysis was performed using LIBSVM software (http://www.csie.ntu.edu.tw/~cjlin/libsvm/). Inputs into the MVPA were the smoothed GM maps computed from the VBM analyses. The MVPA method uses a nonlinear classifier based on support-vector machine algorithms with radial basis function (RBF) kernels. Briefly, at each voxel v i, a 3 × 3 × 3 neighborhood (“searchlight”) centered at v i was defined. The spatial pattern of voxels in this block was defined by a 27-dimensional vector, which was inputted into the classifier. Classifier performance for v i was computed using an M-fold cross-validation procedure. In the M-fold (here M = 10) cross-validation procedure, the data were randomly divided into M-folds. M-1 folds were used for training the classifier, and the remaining fold was used for testing. This procedure was repeated M times wherein a different fold was left out for testing each time. Class labels of the test data were estimated at each fold, and average classification accuracy was computed for each fold, termed cross-validation accuracy (CA). [file 13229_2015_42_MOESM2_ESM.tiff]
